# Immunity protein release from a cell-bound nuclease colicin complex requires global conformational rearrangement

**DOI:** 10.1002/mbo3.122

**Published:** 2013-08-28

**Authors:** Mireille Vankemmelbeke, Nicholas G Housden, Richard James, Colin Kleanthous, Christopher N Penfold

**Affiliations:** 1 School of Life Sciences, Centre for Biomolecular Sciences, University of NottinghamUniversity Park, Nottingham, NG7 2RD, United Kingdom; 2 Department of Biochemistry, University of OxfordSouth Parks Road, Oxford, OX1 3QU, United Kingdom

**Keywords:** Bacteriocin, colicin, disulfide bond, *Escherichia coli*, translocation

## Abstract

Nuclease colicins bind their target receptor BtuB in the outer membrane of sensitive *Escherichia coli* cells in the form of a high-affinity complex with their cognate immunity proteins. The release of the immunity protein from the colicin complex is a prerequisite for cell entry of the colicin and occurs via a process that is still relatively poorly understood. We have previously shown that an energy input in the form of the cytoplasmic membrane proton motive force is required to promote immunity protein (Im9) release from the colicin E9/Im9 complex and colicin cell entry. We report here that engineering rigidity in the structured part of the colicin translocation domain via the introduction of disulfide bonds prevents immunity protein release from the colicin complex. Reduction of the disulfide bond by the addition of DTT leads to immunity protein release and resumption of activity. Similarly, the introduction of a disulfide bond in the DNase domain previously shown to abolish channel formation in planar bilayers also prevented immunity protein release. Importantly, all disulfide bonds, in the translocation as well as the DNase domain, also abolished the biological activity of the Im9-free colicin E9, the reduction of which led to a resumption of activity. Our results show, for the first time, that conformational flexibility in the structured translocation and DNase domains of a nuclease colicin is essential for immunity protein release, providing further evidence for the hypothesis that global structural rearrangement of the colicin molecule is required for disassembly of this high-affinity toxin-immunity protein complex prior to outer membrane translocation.

## Introduction

Nuclease colicins are *Escherichia coli* bacteriocins which have to traverse two membranes in order to gain access to their site of action, the cytoplasm. They bind sensitive cells via the vitamin B_12_ receptor BtuB receptor in the outer membrane (OM) and much progress has been made to unravel the events leading to OM translocation of their cytotoxic domains (Cascales et al. 2007; Kleanthous 2010; Jakes and Cramer 2012). In common with most colicins, the DNase-type colicin E9 (colE9) consists of three functional domains. The killing activity is contained in its C-terminal DNase domain; the central receptor-binding (R) domain binds the BtuB in the OM, while the N-terminal translocation (T) domain engages the cellular energy transducing Tol system in order to achieve OM translocation of its cytotoxic domain. Upon synthesis colE9 forms a high-affinity interaction with its cognate immunity protein, Im9, also encoded by the colicin operon (Kleanthous and Walker 2001), that protects colicin-producing cells against DNA damage and potential suicide prior to the release of the complex in the environment.

**Figure 1:**
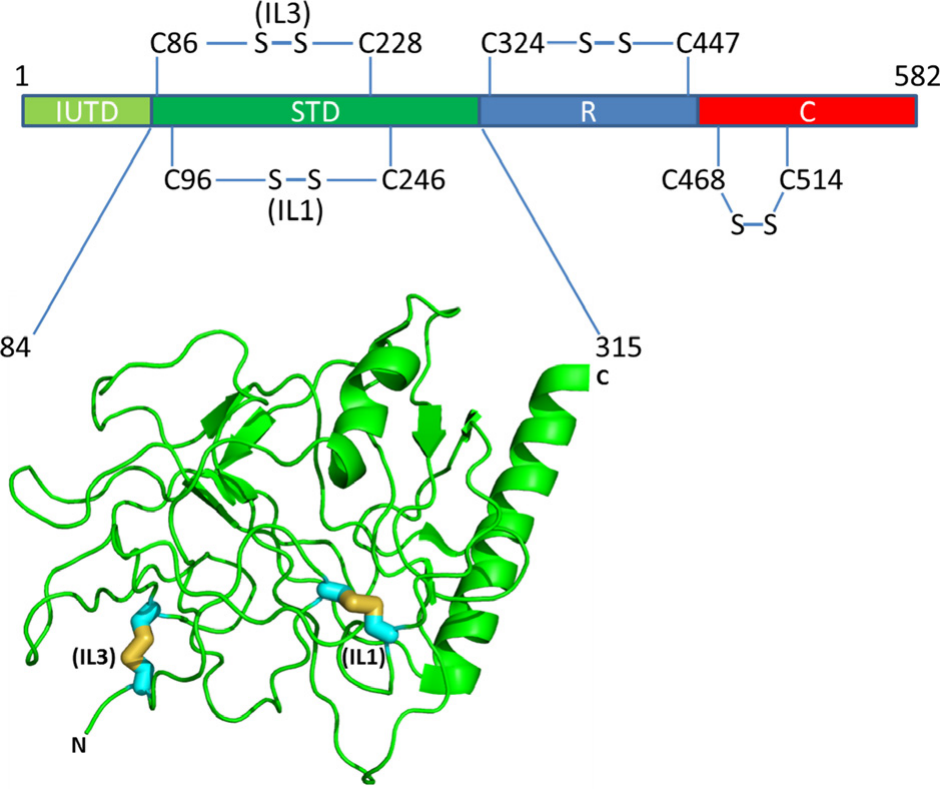
Schematic representation of the domain arrangement of colE9 showing the positions of the disulfide bonds that have been generated in the STD, R, and DNase domains (see Table 1) and the crystal structure of colE3 from residues 84–315 (Soelaiman et al. 2001) that depict the STD with the locations of the corresponding cysteine mutations and consequent disulfide bond formation in the STD of colE9 for IL1 and IL3.

The T domain of colE9 consists of residues 1–315 and has two components: the first 83 residues, commonly referred to as the intrinsically unstructured T domain (IUTD) because of a lack of secondary structure and a large degree of flexibility, contain the OmpF- and TolB-binding sites (Collins et al. 2002; Tozawa et al. 2005; Loftus et al. 2006; Housden et al. 2010), and a globular region or structured T domain (STD) from residues 84–315 that consists of three β-sheets flanked by two helical segments, forming a jellyroll structure (see Fig. 1; Soelaiman et al. 2001). The colE9 IUTD recruits the OM translocator OmpF via a process called “directed epitope delivery” in which two OmpF-binding sites, OBS1 (residues 2–18) and OBS2 (residues 54–63), penetrate the lumen of the OmpF trimer sequentially to access the cell periplasm. The TolB-binding epitope (TBE), sandwiched between OBS 1 and 2, subsequently interacts with TolB to harness the cellular energy in order to promote immunity protein release and cell entry of the cytotoxic domain (Housden et al. 2005, 2010; Yamashita et al. 2008; Bonsor et al. 2009).

Few studies have addressed the role of the STD in colicin translocation. In the RNase colE3 this region participates in the interaction with its immunity protein (Im3) in such a way that Im3 is sandwiched between the RNase and T domain, with 38% of its buried surface contacting the T domain (Soelaiman et al. 2001). It is currently unclear whether a similar scenario exists for colE9, as results from interaction studies with the full-length protein or its isolated DNase domain suggested limited involvement of the T domain in the interaction with Im9 (Wallis et al. 1995). We have previously demonstrated through the engineering of protease cleavage sites in the STD of colE9 that this region remains largely accessible to the extracellular environment in the receptor-bound, disulfide-bonded, colE9/Im9 complex (Zhang et al. 2008).

The nature of the complex formation between colE9 and Im9 and other colicin/immunity complexes has been well characterized, in contrast to the molecular mechanisms that govern the loss of the immunity protein from the colicin complex, a prerequisite for cell entry of the DNase domain. We and others have previously shown that, receptor binding and OmpF recruitment, in isolation, are insufficient to promote immunity protein release (Housden et al. 2005; Zhang et al. 2008). In contrast, translocation of the IUTD of colE3 through OmpF has been claimed to promote Im3 release through a weakening of its binding interface with the T domain (Zakharov et al. 2006, 2008). Additionally, a requirement for the cytoplasmic membrane proton motive force as the energy source for Im9 release from the colE9/Im9 complex was demonstrated, with the Tol proteins being involved in transducing this energy to the OM (Bonsor et al. 2009; Vankemmelbeke et al. 2009).

Unfolding of nuclease colicins to enable cell entry has been inferred from several observations: (i) some loss of helical structure at the distal ends of the 135-residue colE3 receptor-binding domain when bound to BtuB (Kurisu et al. 2003); (ii) the inhibition of colE9 biological activity by an engineered disulfide bond across the extreme end of its receptor-binding domain, which was reversible by dithiothreitol (DTT) treatment (Penfold et al. 2004); (iii) the requirement for the loss of the Im3-translocation domain interaction in colE3-Im3 (Walker et al. 2003); and (iv) a small increase (∼8 Å) in the distance between the translocation and nuclease domains of colE3 upon BtuB or OmpF recruitment, observed by in vitro Fluorescence resonance energy transfer (FRET) analysis (Zakharov et al. 2008). In contrast, analysis of the interaction of the colE9 disulfide-bonded receptor-binding domain with BtuB revealed similar thermodynamic parameters compared to the wild-type protein suggesting that receptor binding alone does not induce gross conformational rearrangement of the colicin receptor-binding domain (Housden et al. 2005).

Although translocation-induced protein unfolding is now generally accepted in the fields of toxin biology and protein secretion, there remains considerable uncertainty about the nature of the unfolding events associated with colicin import and how this impacts on immunity protein release. Recent work by David Brockwell and colleagues have unraveled the paradox that exists between rapid Immunity protein release from the nuclease domains of enzymatic colicins at the cell surface and the perceived astronomical forces needed to achieve this feat (Farrance et al. 2013). Using Atomic Force Microscopy and disulfide bond linkages of the isolated DNase/immunity domains, they showed that rapid E9DNase:Im9 dissociation occurs following the application of only a small force across the complex that generates localized conformational rearrangements leading to changes in the energy landscape of the interaction and destabilization of the complex (Farrance et al. 2013). We show here through the engineering of disulfide-induced rigidity into the STD and DNase domain of colE9 that a global conformational rearrangement of the colicin molecule extending beyond the DNase domain is required in order to release its immunity protein. Additionally, the disulfide bonds in the immunity-free colicin inhibited cell killing activity without impacting on in vitro catalytic activity, suggesting that the unfolding initiated by OM translocation of the colicin IUTD is transduced along the entire molecule in order to achieve cellular uptake of the DNase domain.

## Material and Methods

### Plasmids, bacterial strains, and media

*Escherichia coli* DH5α was used as the host strain for cloning and mutagenesis. *Escherichia coli* BL21 (DE3) (Novagen, Merck Millipore, Darmstadt, Germany) were used as the host strain for the expression vector pET21a (Novagen), which has a strong, IPTG-inducible T7 polymerase promoter and a C-terminal polyhistidine tag (His-tag) to facilitate the purification of overexpressed proteins. The reporter strain *E. coli* DPD1718 has been described previously (Vankemmelbeke et al. 2005). *Escherichia coli* strain LMG194 (Invitrogen Life Technologies, Paisley, U.K.) containing a *lacZ* deletion (F-Δ*lacZ*74 *galE thi rpsL* Δ*phoA* (*Pvu*II) Δ*ara*714 *leu*:Tn10) and transformed with pAG1 was used for the immunity protein release experiments. Plasmid pAG1 is derived from pML261 and contains a 2.4-kb *Eco*RI-*Hin*dIII fragment that encodes the complete *btuB* gene in the vector pUC8 (Koster et al. 1991). All cultures were routinely grown in Luria-Bertani (LB) broth, or on plates of LB agar, supplemented where required with ampicillin (100 μg mL^−1^) or chloramphenicol (30 μg mL^−1^).

All colicin constructs (Table 1) are derived from plasmid pNP69 encoding the colicin E9 structural gene (*ceaI*) and the Im9 immunity gene (*ceiI*) followed by a C-terminal His-tag, under the control of an inducible T7 promoter in pET21a (Penfold et al. 2004). Single cysteine mutations were introduced using the megaprimer mutagenesis approach (Garinot-Schneider et al. 1996) and double cysteine-containing constructs were generated via restriction digest with the unique *Aat*II site within the colicin sequence. Plasmid pBH29 and pKM1 (Table 1) contain two cysteine mutations in the colE9 R and DNase domain, respectively (Mosbahi et al. 2002; Penfold et al. 2004).

**Table 1:** Cysteine-containing colicin E9 constructs

Name	Cysteine mutations	Location of S-S bond	Reference
pBH29	Y324C-L447C	Receptor-binding domain	Penfold et al. (2004)
pIL1	S96C-S246C	Structured T domain	This work
pIL3	A86C-A228C	Structured T domain	This work
pKM1	D468C-E514C	DNase Domain	Mosbahi et al. (2002)

### Protein purification

ColE9/Im9 complexes were purified by metal chelate chromatography with phosphate-buffered saline pH 7.4 (PBS) elution buffer containing 500 mmol/L NaCl and 1 mol/L imidazole. Where necessary, the immunity protein was removed from the colicin complex as previously described (Vankemmelbeke et al. 2012). The free colicin proteins were then refolded by dialysis against PBS containing 1 mmol/L DTT and stored. The labeled immunity protein for the Im9 release experiments was purified and labeled as previously described (Vankemmelbeke et al. 2009).

### Diamide oxidation and DTT reduction

For diamide oxidation, the protein samples were dialyzed overnight against PBS to remove the DTT. Protein samples were then adjusted to 1 mmol/L with diamide and incubated for 30 min in the dark at room temperature, before extensive dialysis against PBS. For reduction, the protein samples were incubated with 10 mmol/L DTT for 1 h at ambient temperature followed by extensive dialysis against degassed PBS. To avoid the possibility of spontaneous oxidation during certain assays, reduced protein was alkylated using iodoacetamide (50 mmol/L, final concentration) followed by extensive dialysis against PBS.

### Colicin activity assays

The *lux* reporter assay for DNA damage was used to test the activity of wild-type and mutant colicin constructs (Vankemmelbeke et al. 2005). All the assays were done as described previously. Gamma values for the luminescence induction by each colicin were calculated and the activities of the mutant proteins presented as a percentage of the wild-type activity (obtained by comparing the luminescence induced by the mutant colicins to that of the wild-type E9 colicin). DNA cleavage assays using linearized pUC18 DNA were performed on free colicin proteins to check for DNase activity as previously described (Wallis et al. 1992).

### Analytical gel filtration

Analytical gel filtration to detect colicin-OmpF and colicin-TolB complex formation was performed on a Superdex 200 10/300 GL column (GE Healthcare Life Sciences, Buckinghamshire, U.K.) equilibrated in 20 mmol/L potassium phosphate, pH 6.5 in the presence of 1% (w/v) n-octyl-β-D-glucopyranoside at 22°C. Protein solutions were buffer exchanged into the gel filtration buffer using a 5 mL HiTrap desalting column (GE Healthcare). Samples of 30 μmol/L colicin, 30 μmol/L TolB, 15 μmol/L OmpF trimer, 30 μmol/L colicin + 30 μmol/L TolB, and 30 μmol/L colicin + 15 μmol/L OmpF were injected as 200 μL aliquots onto the column and eluted at a flow rate of 0.5 mL/min, monitoring the absorbance of the eluate at 280 nm.

### Immunity protein release assay

The assay was performed as previously described (Vankemmelbeke et al. 2009). Briefly*,* wild-type *E. coli* LMG194 (pAG1) from overnight cultures were grown to mid-log (OD_600 nm_ ∼0.4–0.6) in M9 minimal medium containing 0.2% (w/v) glucose and casamino acids. ColE9/Alexa Fluor-labeled Im9 (Im9^AF^) complexes were formed by preincubation of oxidized Im9-free colE9 constructs with Im9^AF^ at a molar ratio of 1:1.5 and were subsequently added to 1 mL of cells in duplicate (10 nmol/L final concentration). The release of Im9^AF^ in the medium after 30 min at 37°C (DTT-treated and control cells [in the absence of reducing agent]) was measured using a Victor^2^ 1420 multilabel plate reader (Wallac, Perkin Elmer Wallac, Buckinghamshire, U.K.), after analysis of the cell-bound relative fluorescence units (RFU). Error bars represent the means ± SEM of three independent experiments.

## Results and Discussion

### Characterization of the disulfide-containing colicin E9 T domain constructs

At present, the extent of unfolding of a receptor-bound nuclease colicin conducive to immunity protein release and the role of the STD in this process are unclear. We decided to investigate these ambiguities by introducing conformational constraint in the STD of the colE9 through disulfide bond engineering and analyzing their effect on Im9 release during OM translocation and on colicin biological activity.

**Figure 2:**
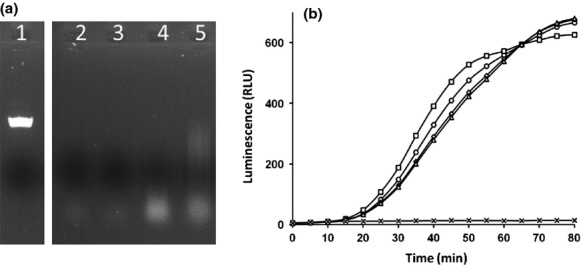
Validation of the in vitro and in vivo DNA cleavage activity of the STD constructs. (A) DNA cleavage activity of the Im9-free STD colicin constructs showing 125 ng of *Nde*I linearized untreated pUC18 (lane 1) and its cleavage by the DNase activity of reduced and alkylated IL1 (lane 2), oxidized IL1 (lane 3), reduced and alkylated IL3 (lane 4), and oxidized IL3 (lane 5). (B) DNA damage-induced luminescence (relative luminescence units [RLU]) in *Escherichia coli* DPD1718 cells by incubation with 4 nmol/L reduced and alkylated IL1 (○), IL3 (♢), KM1 (Δ), BH29 (□), and LB medium (×).

We introduced disulfides at two flexible loop locations in the STD of colE9 (constructs pIL1 and pIL3, Table 1 and Fig. 1). Locations for the introduction of the single cysteine mutations were identified using the coordinates of colE3 (pdb:1JCH, [Soelaiman et al. 2001]) as template for colE9 and the program S-S bond (http://eagle.mmid.med.ualberta.ca/forms/ssbond.html) which selects residues with beta carbons between 3.42 and 4.24 Å apart. Although colE9 has one extra residue (at residue 126 of colE9) over its T and R domains compared to colE3, colE9 and colE3 have 91.8% identity over their STD indicating significant conservation of residues over this region. Those residues chosen with supporting chemistries for disulfide bond formation were Ala96 and Ala245 (Ala246 in colE9), and Ser86 and Ser227 (Ser228 in colE9) according to Table 1. The presence of all cysteine mutations in the colicin constructs was confirmed through DNA sequencing and killing assays on the single cysteine mutants showed that the mutations had no affect on their biological activity (data not shown). The double cysteine-containing colicin constructs were generated via restriction digest and the resulting proteins purified. Disulfide bond formation upon oxidation was confirmed by differential reactivity with the thiol-specific Ellman's reagent of the free oxidized and reduced colicin constructs (data not shown). Size-exclusion chromatography of the oxidized constructs on a Superdex-75 10/300 GL (GE healthcare) column and sodiumdodecyl sulfate polyacrylamide gel electrophoresis (SDS-PAGE) confirmed their predominant monomeric nature (data not shown). In order to verify that the disulfide bonds had not unduly abolished catalytic activity of the nuclease colicin, we performed an in vitro DNA cleavage assay. The results show that the oxidized free constructs cleave DNA as efficiently as the reduced and alkylated proteins (Fig. 2A).

Two previously characterized colE9 constructs, pBH29 and pKM1, contain a disulfide bond in the R and DNase domain, respectively (Table 1), which reversibly affect their biological activity (Mosbahi et al. 2002; Penfold et al. 2004). The biological activity of all four reduced and alkylated constructs (BH29, R domain; KM1, DNase domain and IL1 and IL3, STD) was analyzed using our lux assay for DNA damage (Vankemmelbeke et al. 2005). The two new constructs (IL1 and IL3) and KM1 exhibited between 70% (IL3 and KM1) and 84% (IL1) of the activity of reduced and alkylated BH29 (Fig. 2B), indicating that the introduced cysteine mutations had not compromised biological activity, further confirming the DNA cleavage results.

### Disulfide bonds in the STD and DNase domains inhibit Im9 release from receptor-bound colicin E9/Im9 constructs

Earlier work by our group and colleagues has shown that an input of cellular energy, in the form of the cytoplasmic membrane proton motive force, is required in order to release the Im9 protein from a cell-bound colE9/Im9 complex and initiate OM translocation of the DNase domain (Bonsor et al. 2009; Vankemmelbeke et al. 2009). The underlying structural transitions enabling these two events and the degree of unfolding of the colicin molecule are currently not clearly defined. We envisaged that the energy input would be utilized for the unfolding of the receptor-bound colicin which would destabilize its interaction with the immunity protein, bringing about its expulsion, in preparation for OM translocation of the DNase domain. We decided to test this hypothesis by analyzing the effect of the disulfide bonds in the STD (IL1 and IL3) and DNase domains (KM1) of colE9 on Im9 release. We used the previously characterized construct containing the disulfide in the R domain of colE9 (BH29) as the control in these experiments.

**Figure 3:**
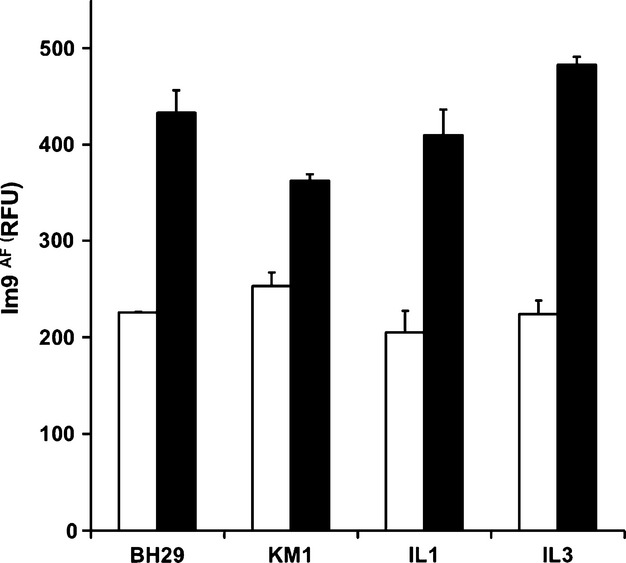
Immunity protein release (Im9^AF^) from oxidized disulfide – containing colE9 constructs. Release of immunity protein following reduction of the disulfide bond is shown as relative fluorescence units (RFU) between *Escherichia coli* LMG194 (pAG1) cells preincubated with 10 nmol/L of each oxidized colicin E9 construct in the presence (black bars) and absence (white bars) of 2 mmol/L DTT after a 30-min incubation period at 37°C.

Disulfide-containing colicin complexes with Alexa Fluor-labeled Im9 protein were preformed (Vankemmelbeke et al. 2009) and incubated with sensitive cells as described in Material and Methods. In the absence of reducing agent, all constructs, including BH29, showed a similar background Im9 release which we have previously attributed to incomplete oxidation of the disulfide-containing constructs (Vankemmelbeke et al. 2005, 2009), giving rise to a small amount of background activity (see Fig. 5) and hence immunity protein release. Addition of DTT led to a twofold increase in Im9 release for all constructs with the exception of KM1 for which the DTT-induced release was somewhat less (Fig. 3). It is possible that the disulfide in cell-bound KM1 is less accessible to DTT reduction; alternatively, the reduced thiols may readily reoxidize in the cell-bound construct (Mosbahi et al. 2002).

This is the first time that flexibility within the STD has been shown necessary for immunity protein release from a nuclease colicin complex despite the low forces required at the N terminus to activate the “trip bond” effect and dissociate immunity from the colicin complex (Farrance et al. 2013). Both disulfides in the STD (IL1 and IL3) have a similar inhibitory effect on Im9 release, in spite of the disulfide in IL3 being closer to the IUTD region than in IL1 and perhaps exhibiting different unfolding characteristics. As outlined in the introduction, there is some ambiguity about the location of Im9 in the colE9/Im9 complex. If Im9 is centered between the DNase and STD, analogous to the orientation of the colicin E3/Im3 complex (Soelaiman et al. 2001), then one could envisage that introducing rigidity into the latter would have a knock-on effect on immunity protein release. Evidence to support this model, however, is currently lacking, as it is equally plausible that Im9 resides on the external side of the colE9/Im9 complex. Intriguingly, Im9 release remains hindered by the disulfide in the DNase domain suggesting that unfolding of the STD and R domains in isolation are insufficient to dislodge the immunity protein, perhaps supporting the latter model. We suggest that a conformational rearrangement requiring a cellular energy input and initiated via the recruitment of the Tol system by the colicin IUTD is propagated along the STD (IL1 and IL3), R domain (BH29), and finally DNase domain (KM1) culminating in the release of the immunity protein. The proximity of the OM surface may also contribute to this process, providing electrostatic attraction of the positively charged DNase and repulsion of the negatively charged immunity protein (Walker et al. 2007; Vankemmelbeke et al. 2012).

### Locked colicin with disulfide bonds in the STD still interact with OmpF and TolB

**Figure 4:**
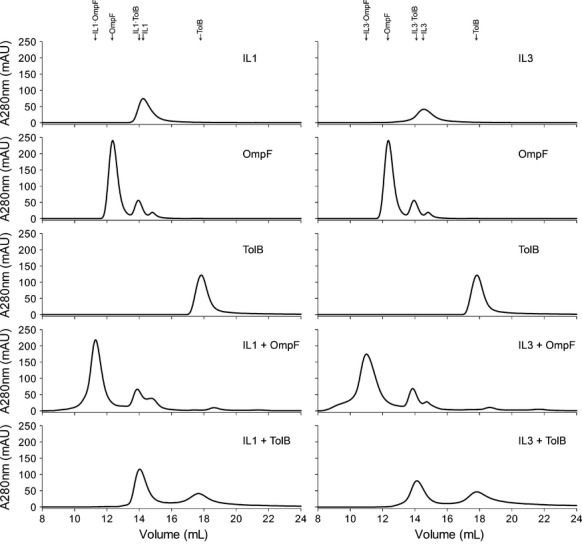
Analytical gel filtration showing oxidized IL1 and IL3 interact with OmpF and TolB. Absorbance (A_280_) measurements over elution volume are shown for IL1 (left panel), IL3 (right panel), with from top to bottom: single IL1/3 constructs, OmpF and TolB, followed by mixtures of IL1/3 constructs with OmpF, and IL1/3 with TolB. A shift in retention time of complexed proteins of IL1::OmpF and IL1::TolB, IL3::OmpF and IL3::TolB (fourth and fifth panels, respectively) is shown at the top of the figure.

Although the biological data above suggests that the disulfide locked IL1 and IL3 proteins interact with BtuB, it is possible that the inactivity of the mutants is due to their inability to bind directly to OmpF and/or TolB per se rather than any inability to bind to OmpF and/or TolB in vivo due to structural frustration inflicted on the colicins by the disulfide bonds. Analytical gel filtration showed that both disulfide locked IL1 and IL3 colicins still had the propensity to interact with both OmpF and TolB in vitro corroborating our conclusions that global conformational rearrangements along the entire colicin are necessary for biological activity (Fig. 4). Conformational flexibility is essential for the passage of the colE9 IUTD through the lumen of the OmpF trimer and formation of the translocon complex (Yamashita et al. 2008). The colE9 translocon involves the simultaneous interaction of the porin-binding sequences, OBS1 and OBS2, of the IUTD with two of the three subunits of OmpF enabling binding of the TolB-TBE with TolB in a fixed orientation (Housden et al. 2013). This sequence of events facilitates the connection of the colicin with the proton motif force, release of Im9, and subsequent cell entry (Vankemmelbeke et al. 2009). It is uncertain to what extent the disulfide bonds in IL1 and IL3, once bound to BtuB, place a conformational restraint on the threading of the IUTD through the OmpF porin even though an interaction with OmpF and TolB is seen in vitro but a disulfide bond engineered into the top of the receptor-binding domain of colE9 did not hinder occlusion of the IUTD by OmpF (Zhang et al. 2008).

### Biological activity is reversibly inhibited in the oxidized colicin E9 constructs, following Im9 removal

Construct BH29 has been used in the past for cell killing synchronization purposes (Penfold et al. 2004; Housden et al. 2005; Zhang et al. 2008; Vankemmelbeke et al. 2009). Im9-free BH29 is inactive when bound to sensitive cells in oxidized form, resuming activity upon reduction of the disulfide bond by DTT. In order to find out whether the disulfide bonds in the Im9-free STD constructs (IL1 and IL3) would prevent biological activity in a similar manner, we analyzed the killing activity of the oxidized constructs in the absence of Im9 using our sensitive lux assay (Vankemmelbeke et al. 2005).

**Figure 5:**
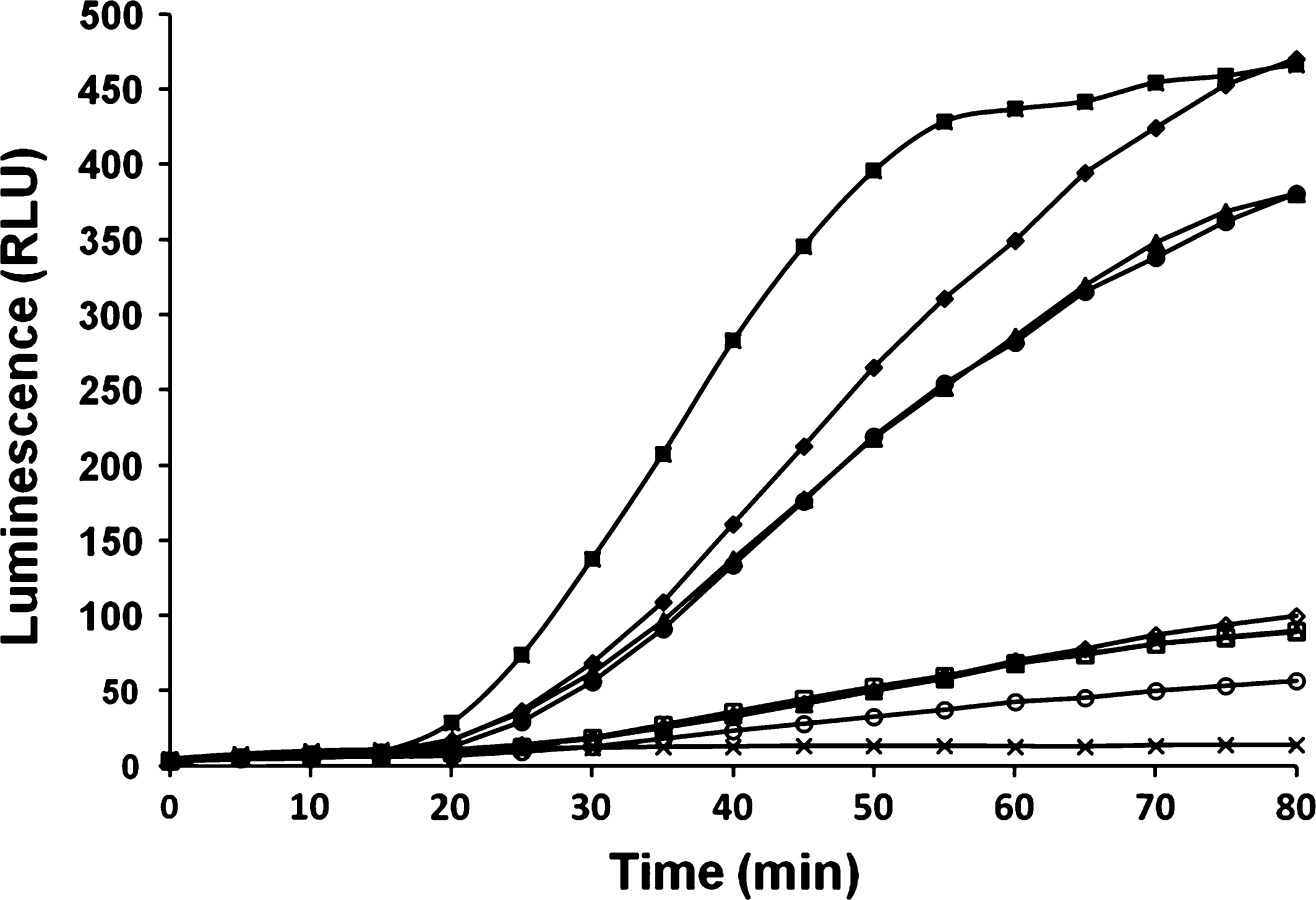
Disulfide bonds reversibly inhibit cell killing by oxidized Im9-free colE9 constructs. The luminescence induced in *Escherichia coli* DPD1718 cells preincubated, in duplicate, with 4 nmol/L oxidized colicin E9 constructs, IL1 (○), IL3 (◊), KM1 (Δ), BH29 (□) and 4 nmol/L oxidized and then reduced colE9 constructs, IL1 (•), IL3 (♦), KM1 (▲), BH29 (▪) following addition of 1 mmol/L DTT after 1 min, or with no addition (x) is shown with time.

Both disulfide bonds in the STD impeded cell killing, which was reversed by the addition of reducing agent, in analogy to the disulfide bond in the R and DNase domain (Fig. 5). All oxidized constructs showed a small amount of background activity which could be due to trace amounts of reduced or multimeric protein in the oxidized protein preparations. The data clearly show that intradomain disulfides in any of the three structural colicin domains inhibit biological activity, consistent with domain unfolding and/or the use of a restricting protein channel being mandatory for immunity release and concomitant colicin OM translocation. This confirms earlier observations by Duché (2007) showing that the colicin E2 R and T domains maintain contact with their binding partners at the OM and periplasmic space, respectively, when the DNase domain gains access to the cytoplasm, an arrangement most likely to result in the unfolding of the colicin (Duché 2007). A recent report on the distantly related *Yersinia pestis* bacteriocin, pesticin, equally established an intricate link between unfolding and cellular import through disulfide bond engineering (Patzer et al. 2012).

## Conclusion

Previously, rigidity engineered into the receptor binding and IUTD domains of colE9 through the establishment of disulfide bonds have prevented immunity release and biological activity of cell-bound colE9 (Penfold et al. 2004; Zhang et al. 2008). We extend these results and show that disulfide bonds produced in the globular translocation and DNase domains similarly prevent immunity release with a concomitant detoxifying effect in vivo. These observations suggest that the unfolding requirement is likely to be a general principle for bacteriocin OM translocation and may provide an explanation for the tendency of bacteriocins to hijack cellular energy transducing systems (Jakes and Cramer 2012). This would enable the toxins to make use of the cellular energy, ATP or the inner membrane-associated proton motive force as a source of free energy to promote mechanical unfolding and membrane translocation (Thoren and Krantz 2011). The recent finding that colicin:immunity protein dissociation occurs at relatively low applied forces (≤20 pN) and within the energy ranges generated by the proton motive force (Farrance et al. 2013; Robinson 2013), provides further evidence for the role of internal cellular energy systems in external transport processes.
